# Biclonal Desmoid-Type Fibromatosis With Two Beta-Catenin Mutations: Evidence for the Recruitment of Normal Myofibroblasts

**DOI:** 10.7759/cureus.28006

**Published:** 2022-08-14

**Authors:** Keith M Skubitz, Paari Murugan, Christopher L Corless

**Affiliations:** 1 Department of Medicine, University of Minnesota, Minneapolis, USA; 2 Department of Laboratory Medicine and Pathology, University of Minnesota, Minneapolis, USA; 3 Department of Pathology and Knight Cancer Institute, Oregon Health & Science University, Portland, USA

**Keywords:** tumor microenvironment, bi-clonal, clonality, sarcoma, beta-catenin, ctnnb1, fibromatosis, desmoid

## Abstract

Sporadic aggressive fibromatosis, or desmoid-type fibromatosis, is characterized by oncogenic mutations in CTNNB1. The clonal cell is a myofibroblast-like cell, and it has been hypothesized that the recruitment of normal myofibroblasts could contribute significantly to the tumor. We describe a case in which a CTNNB1 p.T41A mutation was present at a mutant allele frequency of 30%, suggesting that a significant proportion of the tumor myofibroblasts may have been recruited from normal precursor pools. In addition, a small subclone with a p.S45F mutation (allele frequency of 2%) was identified in the tumor. This case provides additional evidence that myofibroblasts recruited by a tumor from a normal precursor pool contribute significantly to the tumor; such recruitment could impact response to treatment and long-term outcomes.

## Introduction

Aggressive fibromatosis, also known as a desmoid tumor or desmoid-type fibromatosis (DTF), is a clonal, locally invasive tumor with a highly variable natural history that does not metastasize [1-4]. Histologically, DTF appears as a poorly circumscribed proliferation of myofibroblast-like cells with variable collagen deposition, which exhibits morphological intra- and inter-tumoral heterogeneity [5-7]. DTF is a true clonal neoplasm [8] in which the Wnt (beta-catenin) pathway plays an important role. Loss of function mutations in the APC gene is present in those cases associated with familial adenomatous polyposis. In sporadic DTF, most cases have a mutation in CTNNB1, the gene encoding beta-catenin, although cases of APC mutations have rarely been found [2-4,9]. In one study of 254 sporadic DTF cases, 223 (87.8%) had one of three CTNNB1 mutations identified: p.S45P, p.S45F, or p.T41A; less than 10% harbored the p.S45P mutation [10]. Some studies have found that the CTNNB1 p.S45F mutation correlates with a higher risk of recurrence compared with the other mutations, although not all reports have confirmed this observation [2,4,11-16]. One study found a higher rate of progression arrest on imatinib treatment as compared with tumors having wild-type CTNNB1 [17].

One model of DTF pathogenesis proposes that an activating stimulus in the setting of dysregulation of beta-catenin leads to the up-regulation of beta-catenin, nuclear translocation of beta-catenin, increased Wnt target gene expression, cell growth, and extracellular matrix (ECM) production [2,4]. Associated signaling molecules may also recruit normal nonclonal myofibroblast precursor cells, such as profibrotic ADAM12-positive cells from a PDGFR-positive precursor pool [4,6,18], thus adding normal nonclonal myofibroblasts to the tumor.

We report a case of DTF having two different CTNNB1 mutations in which next-generation sequencing also suggested a significant contribution of normal myofibroblast-like cells to the tumor.

## Case presentation

**Figure 1:**
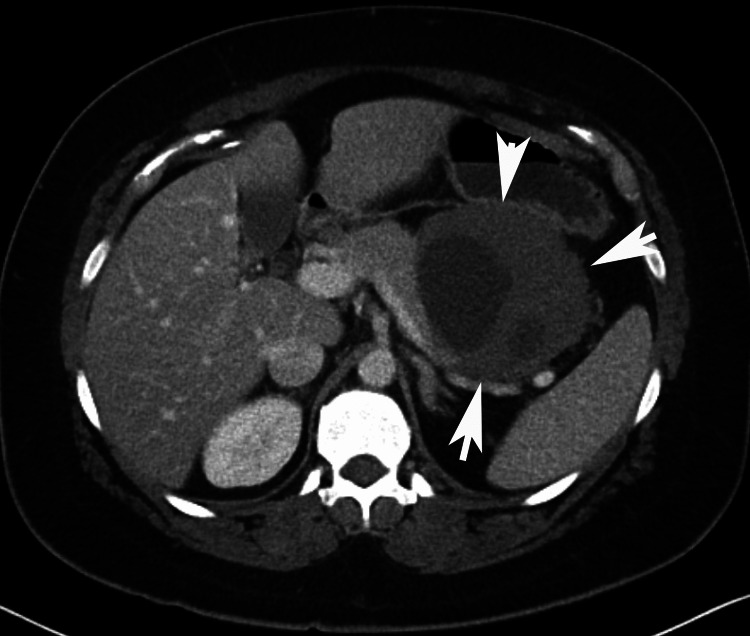
CT image at presentation showing the large tumor The tumor is indicated by white arrowheads.

A 39-year-old woman developed sudden-onset fever, chills, and severe abdominal pain. She had been on birth control pills for many years but stopped them two months before her presentation. Past history was notable for obesity, no drug or chemical exposure, and a congenital sixth finger on the left hand that was removed at age 2. The family history was non-contributory. She was never a smoker. Imaging showed an abdominal mass with possible free air (Figure 1), and an exploratory laparotomy revealed a perforated abdominal mass with intra-abdominal abscess and peritonitis. 

**Figure 2:**
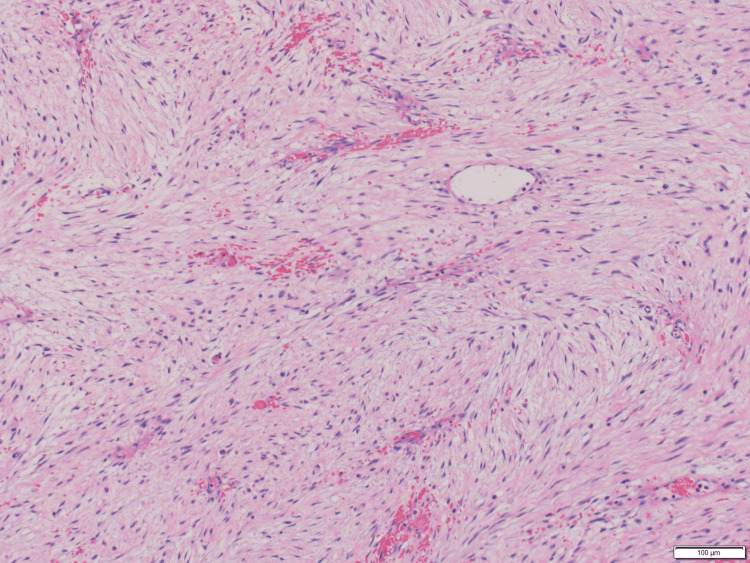
Histologic examination of the resection specimen Typical histologic features of desmoid fibromatosis characterized by sweeping fascicles of bland appearing spindled cells with elongated nuclei and indistinct cytoplasmic borders. Slit-like blood vessels and extravasated erythrocytes are also present. H&E x100

**Figure 3:**
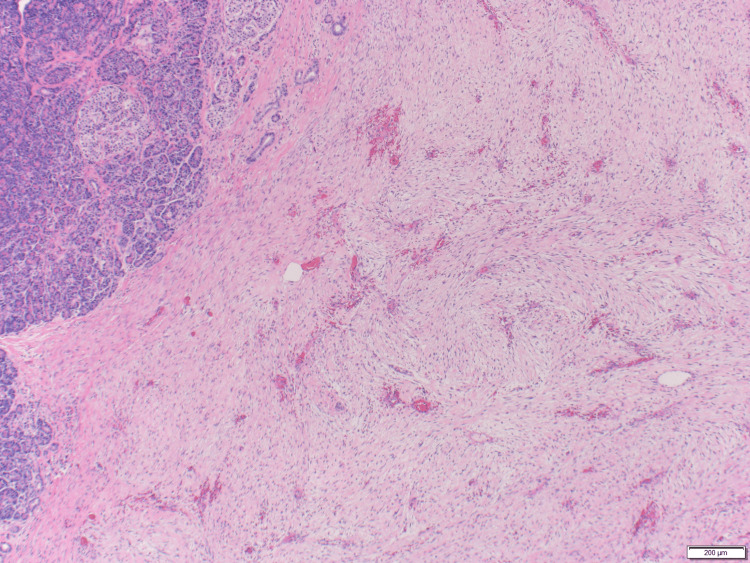
Desmoid fibromatosis (right) invading the pancreatic parenchyma (left) H&E x40

**Figure 4:**
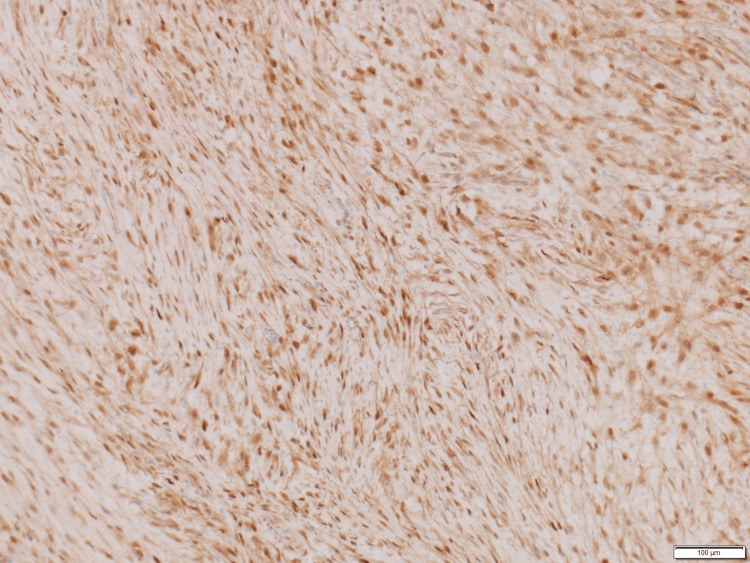
Beta-catenin immunohistochemistry demonstrating nuclear and cytoplasmic staining in the tumor cells Immunohistochemistry (IHC) x100

She underwent an en-bloc resection of an upper abdominal mass with partial colectomy, partial gastrectomy, distal pancreatectomy, and splenectomy. Pathology revealed desmoid type fibromatosis (DTF) involving the omentum, pancreas, and the walls of the stomach and colon with negative margins of resection (Figures 2-4).

An MRI of the pelvis two months after surgery was consistent with intramural fibroids. A biopsy of a uterine mass seven months after the original surgery was benign. Follow-up imaging 16 months after primary surgery showed no recurrence of DTF, a stable uterine leiomyoma, and hepatic steatosis.

Beta-catenin mutation testing was performed to detect alterations in APC and CTNNB1. Each specimen was examined microscopically, and genomic DNA was extracted from dissected, tumor-rich areas. Mutations were screened by targeted massively parallel sequencing using a combination of multiplexed PCR and sequencing on an Illumina platform (GeneTrails® Solid Tumor Panel, Knight Diagnostics Laboratories, Portland, Oregon). The panel covers target exons and portions of flanking intronic sequences of 225 cancer-related genes, including CTNNB1 and APC.

On microscopic review, the estimated tumor fraction in the specimen was 90%. Sequencing to an average read depth of 876 per targeted gene region revealed a CTNNB1 p.T41A mutation at an allele frequency of 30%, and a p.S45F mutation at an allele frequency of 2.4%. The two mutations were on different alleles (in trans). Because the limit of detection of the GeneTrails® panel is a 2% mutant allele, the sequencing was repeated to confirm the p.S45F. The average read depth on the second run was 2684, and nearly identical mutant allele frequencies were observed: p.T41A at 30% and p.S45F at 2.2%. The CTNNB1 copy number was within the normal range for the assay (1.93-1.98 copies in the two runs). No mutations were identified in the APC gene in either run.

Germline genetic testing using the Invitae platform was ordered by the patient’s primary care team to look for familial risk factors. This revealed no mutations in the following genes: APC, ATM, AXIN2, BARD1, BMPR1A, BRCA1, BRCA2, BRIP1, CDH1, CDK4, CDKN2A(p14ARF), CDKN2A (p16INK4), CHEK2, CTNNA1, DICER1, EPCAM, GREM1, HOXB13, KIT, MEN1, MLH1, MSH2, MSH3, MSH6, MUTYH, NBN, NF1, NTHL1, PALB2, PDGFRA, PMS2, POLD1, POLE, PTEN, RAD50, RAD51C, RAD51D, SDHA, SDHB, SDHC, SDHD, SMAD4, SMARCA4, STK11, TP53, TSC1, TSC2, and VHL.

## Discussion

This case provides two important observations. First, substantially less than 50% of the CTNNB1 alleles in the tumor exhibited a mutation, suggesting a significant contribution to the tumor from infiltrating normal myofibroblasts. Second, the demonstration of two beta-catenin mutations suggests the existence of two clonal populations of pathologic DTF cells in the tumor, one having a p.T41A mutation and one a p.S45F mutation.

Beta-catenin is part of the WNT-signaling pathway and plays an important role in cell-cell adhesion. WNT-signaling increases cytoplasmic free beta-catenin, resulting in increased nuclear translocation of beta-catenin, where it functions as a transcriptional co-activator with TCF/LEF and other molecules and thereby alters the expression of WNT target genes [19-20]. Beta-catenin also binds the intracellular domain of E-cadherin and alpha-catenin, which binds actin, thus regulating E-cadherin-mediated cell-cell adhesion and the cytoskeleton [21]. Beta-catenin is regulated by a destruction complex in which phosphorylation of Axin-bound beta-catenin by CK-1 and GSK-3 marks beta-catenin for ubiquitination and proteasomal degradation.

CTNNB1 mutations occur in several tumors, including hepatocellular carcinoma (HCC), endometrial carcinoma, colon cancer, melanoma, and DTF [16,19,22]. The mutations most commonly occur in exon 3 (amino acid residues 5-80), which encodes the highly unstructured and flexible N-terminal domain. Mutations in exon 3 result in a loss of serine/threonine phosphorylation sites and in some cases mutations in the beta-Trcp binding site of the ubiquitin ligase, which decreases beta-catenin ubiquitination and leads to decreased degradation of beta-catenin [16,19]. To date, most DTF samples have been found to contain a p.S45F, p.T41A or p.S45P CTNNB1 mutation. In one study of 33 primary DTF, Colombo et al. suggested that the tumors could be divided into two distinct molecular subgroups with regard to beta-catenin stability, alpha-catenin affinity, and gene expression profiling: tumors with p.T41A or p.S45F mutations versus CTNNB1 wild-type tumors. The gene expression profile of CTNNB1 wild-type DTF was more similar to normal tissues [16]. In addition, the mutated beta-catenins were more stable than the wild-type protein and bound alpha-catenin more weakly [16]. Another study comparing DTFs with normal tissues also identified two distinct subgroups on the basis of gene expression profiles, with potentially different clinical outcomes [7], and suggested a different CTNNB1 mutation distribution between the subgroups [6]. However, not all studies of Wnt target gene expression in DTF have shown differences between wild-type CTNNB1 and the p.T41A or p.S45F mutations [23].

Many studies have tried to correlate the beta-catenin mutation state with clinical behavior. In a large study with 138 patients, Lazar found that recurrence-free survival after surgery was lower in p.S45F mutants (23%) compared to p.T41A mutants (57%) or wild-type (65%) cases [13]. This finding is consistent with several other studies that have found the p.S45F mutation to be associated with worse recurrence-free survival, although not all groups have confirmed this [2,4,11-16]. Koike et al. found the proportion of DTF cases staining strongly for beta-catenin in the cytoplasm rather than in the nucleus was higher in cases with p.T41A than those with p.S45F or wild-type, possibly suggesting less effect on gene transcription with p.T41A than p.S45F [5]. Hamada et al. reported poorer responses to the cyclooxygenase-2 inhibitor, meloxicam, among patients with the p.S45F mutation than those with the p.T41A or p.S45P mutation; however, there was only one case of p.S45P in this study [24]. Nishida et al. did not find a correlation between beta-catenin mutation and response to methotrexate and vinblastine, although the number of patients in each group was small [25]. Kasper found a higher progression arrest rate with imatinib in patients with mutated CTNNB1 compared with wild type [17]. Thus, most studies have suggested that DTF with a p.S45F mutation is more clinically aggressive. A recent large retrospective multi-institutional study found no significant impact of mutation subtype on the efficacy of chemotherapy or tyrosine kinase inhibitors, although there was a trend toward worse outcomes with an APC mutation [26].

CTNNB1 mutations in DTF are similar to those found in other tumors. In HCC and hepatocellular adenoma (HCA), different beta-catenin mutations correlated with different levels of beta-catenin activity [27]. Mutations in p.S45, p.K335, and p.N387 led to weak beta-catenin activation; p.T41 mutations led to moderate activation, and mutations in the beta-TRCP binding site (p.D32-S37) led to high levels of activation [27]. In most HCC cases with beta-catenin mutations in p.S45, the mutant alleles were duplicated, resulting in high beta-catenin activity [27]. Some studies have reported biallelic mutations of CTNNB1 in cancer, including parathyroid adenoma [28], colorectal adenomas [29-30], and some hepatocellular carcinomas [27]. Of 869 colon cancers, 27 had CTNNB1 mutations; among these, 74% were homozygous or hemizygous mutations, suggesting that in colon cancer, a higher threshold of beta-catenin stabilization is needed for transformation than in extra-colonic tumors [22].

Rebouissou identified five cases of HCC with weak or atypical beta-catenin mutations in which a second beta-catenin mutation was present that was predicted to result in high or moderate activation; in three of the five cases, the two mutations were shown to be on the same allele. Doyen et al. reported two patients with more than one DTF lesion [31]; in one case with four distinct DTF lesions, one expressed p.T41A, one expressed both p.S45P and p.S45A, and two wild-type CTTNB1. In the other case, one DTF lesion harbored p.S45P and the other p.T41A. In Case 1, there was a one-year interval between the first and subsequent three lesions, while in Case 2, there was a one-year interval between the two lesions. Thus, there is a precedent in HCC and DTF for two mutations in the same CTTNB1 allele, as well as allele duplication. In our case, we determined that the two mutations were on different alleles. While it is likely that they represent two different clones in the same tumor, this could only be determined by single-cell sequencing, a technique that requires fresh tumor tissue. 

**Figure 5:**
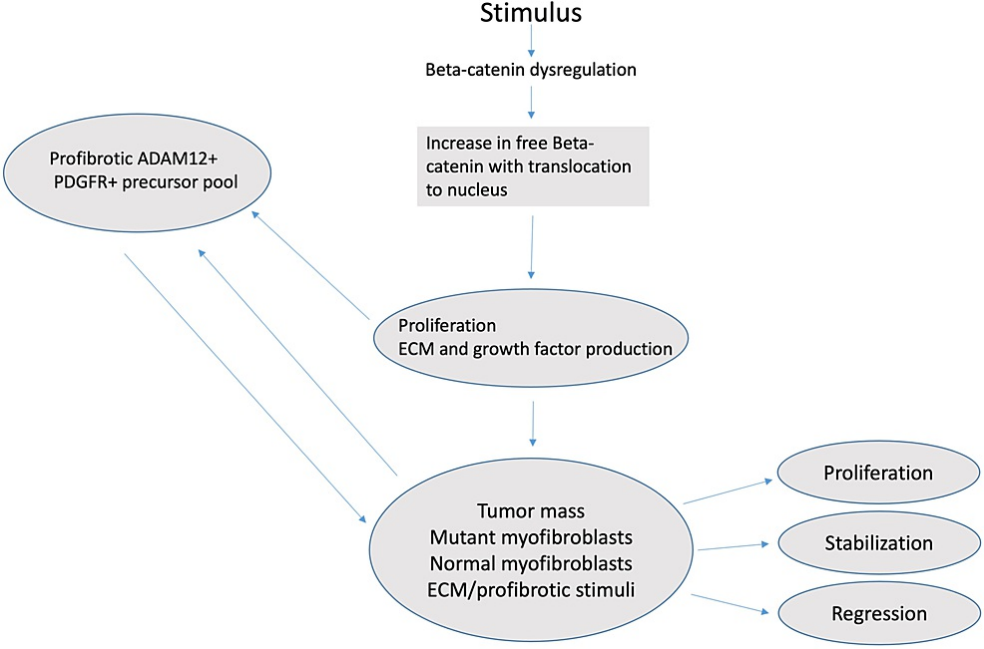
A proposed model of DTF pathogenesis An activating stimulus, with associated inflammation and growth factor production in the setting of deregulated beta-catenin, leads to the up-regulation of beta-catenin. Beta-catenin then translocates to the nucleus where it alters transcription, resulting in a pro-survival signal, growth stimulation, and production of ECM proteins and possibly other growth factors. Factors produced may both further stimulate CTNNB1 mutant myofibroblasts and recruit non-mutant myofibroblasts from a pool of normal precursors, thus adding a non-clonal myofibroblast population to the tumor. The resulting tumor may progress or in some cases, may stabilize or regress. The role of recruited normal myofibroblasts is unknown.

The issue of what percentage of myofibroblastic cells in the tumor harbor a CTNNB1 mutation is of interest. Given that the CTNNB1 copy number was normal (diploid) in the tumor, and assuming that the two CTNNB1 mutations were heterozygous and carried by different clones (totaling ~32% of CTNNB1 alleles), 64% of the cells in the lesion harbored a mutation. Morphologically, the estimated tumor cellularity was 90% so a significant component of the cells that histologically appeared as tumor cells were likely infiltrating normal myofibroblasts, possibly recruited from the pool of ADAM12 (+), PDGFR (+) precursors (Figure 1) [4,6,18]. It will be of interest to determine whether the degree of infiltration of non-mutated myofibroblasts correlates with tumor biology and response to treatment in large prospective studies, preferably in which gene expression studies are also done. Another question of interest is whether there is differential recruitment of non-mutated myofibroblasts based on mutation type. Such studies will require using a technique that allows quantitation of mutant and wild-type CTNNB1. Figure 5 shows a proposed model of DTF pathogenesis.

.

## Conclusions

This case provides two important observations. First, substantially less than 50% of the CTNNB1 alleles in the tumor exhibited a mutation, providing evidence that myofibroblasts recruited by the tumor from a normal precursor pool contributed significantly to the tumor; such recruitment could impact response to treatment and long-term outcome. It will be of interest to determine whether the degree of infiltration of non-mutated myofibroblasts correlates with tumor biology and response to treatment in large studies. Second, the demonstration of two beta-catenin mutations suggests the existence of two clonal populations of pathologic DTF cells in the tumor.
